# Peripheral Inflammation Regulates CNS Immune Surveillance Through the Recruitment of Inflammatory Monocytes Upon Systemic α-Synuclein Administration

**DOI:** 10.3389/fimmu.2019.00080

**Published:** 2019-01-29

**Authors:** Javier María Peralta Ramos, Pablo Iribarren, Luc Bousset, Ronald Melki, Veerle Baekelandt, Anke Van der Perren

**Affiliations:** ^1^Departamento de Bioquímica Clínica, Facultad de Ciencias Químicas, Centro de Investigación en Bioquímica Clínica e Inmunología (CONICET), Universidad Nacional de Córdoba, Córdoba, Argentina; ^2^Institut François Jacob (MIRCen), CEA and Laboratory of Neurodegenerative Diseases, CNRS, Fontenay-aux-Roses, France; ^3^Laboratory for Neurobiology and Gene Therapy, Department of Neurosciences, KU Leuven, Leuven, Belgium

**Keywords:** inflammation, alpha-synuclein, inflammatory monocytes, Parkinson's disease, synucleinopathies

## Abstract

Innate immune activation and chronic neuroinflammation are characteristic features of many neurodegenerative diseases including Parkinson's disease (PD) and may contribute to the pathophysiology of the disease. The discovery of misfolded alpha-synuclein (αSYN) protein aggregates, which amplify in a “prion-like” fashion, has led us to consider that pathogenic αSYN might be hijacking the activation and mobilization mechanism of the peripheral immune system to reach and disseminate within the CNS. Furthermore, our lab and other groups have recently shown that αSYN can adopt distinct fibril conformations or “strains” with varying levels of pathogenic impact. Therefore, the aim of this study was to assess the impact of peripheral inflammation on αSYN spreading in order to better understand the participation of the immune system in the progression of PD. The results presented here show that intraperitoneal LPS injection prior to systemic intravenous recombinant administration of two different αSYN pathogenic strains (fibrils or ribbons) in wild type mice, induces an increase in brain resident microglia and promotes the recruitment of leukocytes toward the brain and the spinal cord. Our findings show for the first time that αSYN can be internalized by LPS-primed inflammatory monocytes, which in turn favors the dissemination from the periphery toward the brain and spinal cord. Further, we found a differential recruitment of CD4^+^ and CD8^+^ T cells after LPS priming and subsequent administration of the αSYN ribbons strain. Together, these data argue for a role of the peripheral immune system in αSYN pathology.

## Introduction

Immunological surveillance of the central nervous system (CNS) has shown to be dynamic, specific, and tightly regulated. Innate immune activation and chronic neuroinflammation are characteristic features of many neurodegenerative diseases including Parkinson's disease (PD) and may contribute to the pathophysiology of the disease (1). During neurodegeneration, peripheral immune cells can gain access to the brain parenchyma (2). Brain-resident microglia encounter myeloid immune cells that have been previously primed in the periphery, establishing an interplay that aggravates the inflammatory process and potentiates neuropathology (3, 4). The recent discovery of a CNS dural lymphatic system that drains macromolecules from the CNS into cervical lymph nodes, further challenges the established basic assumptions of the CNS as an immune privileged site (5–7). Systemic injection of the endotoxin LPS has been widely used as an inflammatory model (8, 9). These peripherally applied stimuli lead to a cytokine-storm that signals to the brain, triggering an immune response. The discovery of misfolded αSYN protein aggregates with different structural characteristics, that could account for the distinct pathological traits within synucleinopathies and which amplify in a “prion-like” fashion (10–14), has led us to consider that pathogenic αSYN might be hijacking the activation and mobilization mechanism of the peripheral immune system to reach and disseminate within the CNS. Therefore, we assessed the impact of peripheral inflammation on αSYN spreading in order to understand the participation of the immune system in αSYN pathology.

## Materials and Methods

### Animals and LPS/αSYN Administration

All animal experiments were carried out in accordance with the European Communities Council Directive of 24 November 1986 (86/609/EEC) and approved by the Bioethical Committee of the KU Leuven (Belgium). Eight-week old female C57BL/6 mice (Janvier, France) were housed under a normal 12 h light/dark cycle with free access to pelleted food and tap water. All surgical procedures were performed using aseptic techniques.

Mice were treated with either (a) 20 μg of LPS i.p., (b) 5 μg of atto-488-labeled pathogenic αSYN fibrils or ribbons i.v. (15), (c) LPS combined with αSYN as aforementioned, or (d) saline, following the administration scheme depicted in **Figure 2A**. Twelve hours after the last injection, mice were euthanised and immune cells were isolated from either whole brain or spinal cord and stained for subsequent flow cytometric analysis. Results are representative of two independent experiments combined (*n* = 3–4 animals per group).

LPS from *Escherichia coli* 055:B5 (purified by gel filtration chromatography) was purchased from Sigma-Aldrich and freshly dissolved in sterile saline prior to i.p. injection. Recombinant αSYN fibrils and ribbons were generated, extensively characterized and labeled with the aminoreactive fluorescent dye atto-488 (ATTO-Tech GmbH) as previously described (13, 15).

### Isolation of Immune Cells From Mice Brains and Spinal Cords

Twelve hours after the last injection, mice were weighed and deeply anesthetized with a ketamine (60 mg/kg, Pfizer)/medetomidine (0.4 mg/kg, Pfizer) cocktail according to their weight. Immune brain cells were isolated from whole brain or spinal cord homogenates as follows. Briefly, mice were transcardially perfused with ice-cold PBS (Gibco) and brains or spinal cords were collected in DMEM (Gibco) supplemented with sodium pyruvate (Gibco) and a penicillin, streptomycin and glutamine cocktail (Gibco), gently disaggregated mechanically and resuspended in PBS containing 3 mg/mL collagenase D (Roche Diagnostics) plus 10 μg/mL DNAse (Sigma-Aldrich) for an enzymatic homogenization. After this incubation, brain homogenates were filtered in 40 μm pore size cell strainers (BD Biosciences), centrifuged 8 min at 1,800 r.p.m., washed with PBS and resuspended in 6 mL of 38% isotonic Percoll® (GE Healthcare) before a 25 min centrifugation at 800 G with 0 acceleration and 0 brake. Myelin and debris were discarded. Cell pellets containing total brain immune cells were collected, washed with DMEM supplemented with 10% fetal bovine serum (Gibco) and cell viability was determined by trypan blue exclusion using a Neubauer's chamber. Finally, cells were labeled for subsequent flow cytometric analysis.

### Flow Cytometric Analysis

Surface staining of single-cell suspension of isolated brain immune cells was performed using standard protocols and analyzed on a FACSCanto II (BD Biosciences). Flow cytometric analysis was defined based on the expression of CD11b, CD45, Ly6C, CD4, and CD8 as follows: microglial cells, CD11b^+^ CD45^lo^; recruited leukocytes, CD11b^+/−^ CD45^hi^; inflammatory monocytes, CD11b^+^ CD45^hi^ Ly6C^hi^; T cells, CD11b^−^ CD45^hi^ CD4^+^/CD8^+^. Data analysis was conducted using FCS Express (*De Novo* Software). The following antibodies were used in the procedure: monoclonal anti-mouse CD11b APC (BioLegend, clone M1/70), CD11b FITC (BD Pharmingen, clone M1/70), CD45 APC-Cy7 (BioLegend, clone 30-F11), Ly6C PE-Cy7 (BD Pharmingen, clone AL-21), CD4 APC (BD Pharmingen, clone RM4-5), CD8 PE (BD Pharmingen, clone 53-6.7) or isotype control antibodies (BD Pharmingen, APC, clone R35-95; PE-Cy7, clone G155-178). Multiparametric gating analysis strategy was performed as previously described (8).

### Statistical Analysis

Results are expressed as mean ± s.e.m. All statistical analyses were performed using Prism® 7.0 (GraphPad Software). Means between groups were compared with one-way analysis of variance followed by a Tukey's *post-hoc* test. Statistical significance levels were set as follows: ^*^/# if *p* < 0.05, ^**^/## if *p* < 0.01, and ^***^/### if *p* < 0.001. The asterisks indicate the comparison against the saline treated group.

## Results and Discussion

**Figure 1:**
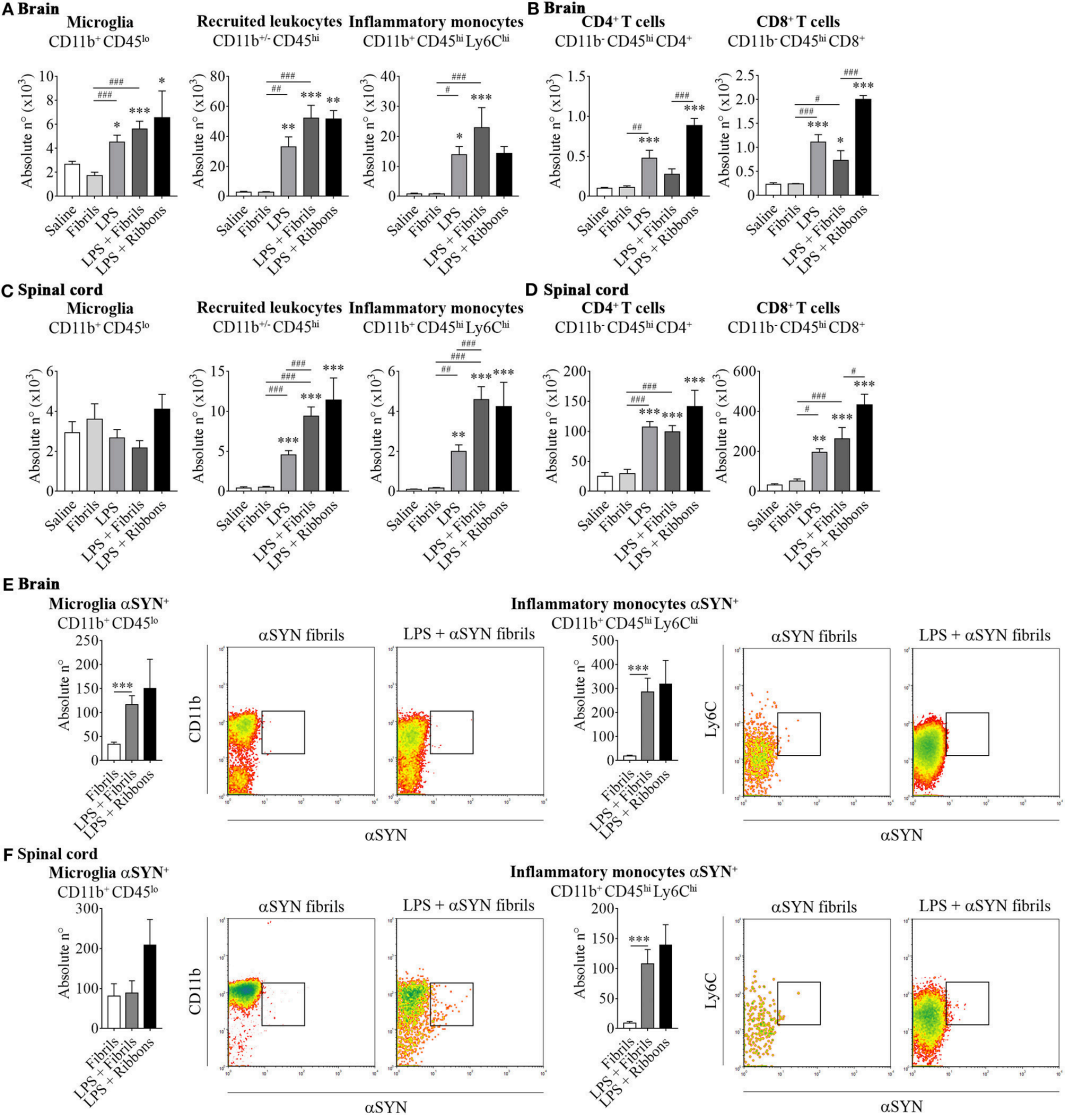
αSYN hijacks the activation and mobilization mechanism of LPS-primed peripheral inflammatory monocytes to disseminate into the CNS. Mice were treated with either (a) 20 μg of LPS i.p (LPS group), (b) 5 μg of atto-488-labeled pathogenic αSYN fibrils or ribbons i.v. (αSYN group), (c) LPS combined with αSYN strains (LPS + αSYN group), or (d) saline alone, following the administration scheme depicted in Figure 2A. Twelve hours after the last injection, mice were euthanised and immune cells were isolated from either whole brain **(A,B)** or spinal cord **(C,D)** and stained for subsequent flow cytometric analysis. Absolute numbers of CD11b^+^ CD45^lo^ microglial cells, CD11b^+/−^ CD45^hi^ recruited cells, CD11b^+^ CD45^hi^ Ly6C^hi^ inflammatory monocytes, CD11b^−^ CD45^hi^ CD4^+^ and CD11b^−^ CD45^hi^ CD8^+^ lymphocytes were assessed by flow cytometry. Absolute numbers of αSYN-internalized CD11b^+^ CD45^lo^ microglial cells or CD11b^+^ CD45^hi^ Ly6C^hi^ inflammatory monocytes purified from brain **(E)** or spinal cord **(F)**, were assessed by flow cytometry. Results are representative of two independent experiments combined (*n* = 3–4 animals per group). Representative CD11b vs. αSYN and Ly6C vs. αSYN density-plots illustrate the gating analysis strategy employed for microglial cells and inflammatory monocytes, when gated in CD45^lo^ or CD45^hi^ cells respectively. Data are expressed as mean ± s.e.m. Means between groups were compared with one-way analysis of variance followed by a Tukey's *post-hoc* test. Statistical significance levels were set as follows: ^*^^/#^ if *p* < 0.05, ^**^^/##^ if *p* < 0.01, and ^***^^/###^ if *p* < 0.001. The asterisks indicate the comparison against the saline treated group.

The results presented here show that intraperitoneal LPS injection combined with intravenous administration of two different recombinant αSYN pathogenic strains (fibrils or ribbons) in wild type mice, induces an increase in brain resident microglia and promotes the recruitment of leukocytes toward the brain (Figure 1A) and the spinal cord (Figure 1C). When further characterizing the phenotypic traits of the peripheral cells trafficking to the CNS, we identified neutrophils and professional antigen presenting dendritic cells among innate myeloid leukocytes (data not shown), as well as a distinct migration of CD4^+^ and CD8^+^ T cell subsets after administration of αSYN strains, which was most prominent in the brain for ribbons compared to fibrils (Figures 1B,D). Moreover, LPS-primed inflammatory monocytes proved to be the major source of CNS-associated phagocytes after systemic challenge with αSYN strains. In the brain, fibrils induced a stronger response compared to ribbons, while the effect in the spinal cord was similar for both αSYN strains (Figures 1A,C). Interestingly, we noticed that LPS priming favored αSYN spreading toward the brain and spinal cord, as observed by an upregulation of αSYN^+^-expressing microglia and inflammatory monocytes (Figures 1E,F).

Mounting evidence supports the notion that the innate immunity has a great capacity of adapting and deploying an innate immune memory upon an inflammatory insult (16, 17), shaping subsequent immune responses in the brain (18). Our findings clearly demonstrate that priming with LPS prior to systemic αSYN challenge, induces an increase in the absolute number of the brain-resident microglia and promotes the recruitment of peripheral leukocytes into the CNS. Similar to other reports (19–21), our results demonstrate that stimulation with LPS of innate immune receptors, such as Toll-like receptor 4, amplifies the inflammation within the CNS.

Sacino and colleagues were the first to describe αSYN pathology in the brain and spinal cord induced by a single peripheral intramuscular injection of αSYN (22). Shortly after that, our group described passage of the blood-brain barrier by recombinant αSYN aggregates and distribution throughout the CNS after systemic administration (13). Our findings show for the first time that αSYN can be internalized by LPS-primed inflammatory monocytes, which in turn favors the dissemination from the periphery toward the brain and spinal cord. In line with our results, Harms et al. (23) described a recruitment of immune cells toward the CNS prior to neurodegeneration after intracranial αSYN fibril treatment. Additionally, peripheral monocyte entry was recently reported to be required for viral vector-mediated αSYN-induced neuroinflammation and neurodegeneration (24). Together, these data argue for a role of the peripheral immune system in αSYN pathology.

Further, we also found a differential recruitment of CD4^+^ and CD8^+^ T cells after LPS priming and subsequent administration of αSYN ribbons compared to fibrils, which was most prominent in the brain. By presenting a different T cell response toward distinct αSYN strains we show the importance of the protein conformation in the capacity to act as an antigenic epitope. Related to this, Sulzer and co-workers recently discovered that PD patient-derived T cells are able to recognize well-defined αSYN peptides (25). The lymphocyte activation gene-3 (LAG3) has been proposed to bind pathogenic αSYN assemblies and to favor their endocytosis and transmission (26). Since effector and regulatory T cells also express LAG3, it will be important to better comprehend the role of this membrane protein since it executes dual roles in autoimmunity and cancer. In this regard, while LAG3 has been shown to play a protective role in autoimmunity by dampening T helper cell responses and promoting regulatory T cell-mediated suppression, it has also been described to bear co-inhibitory features, becoming a target for immune blockade to empower anti-tumor T cell responses (27).

**Figure 2:**
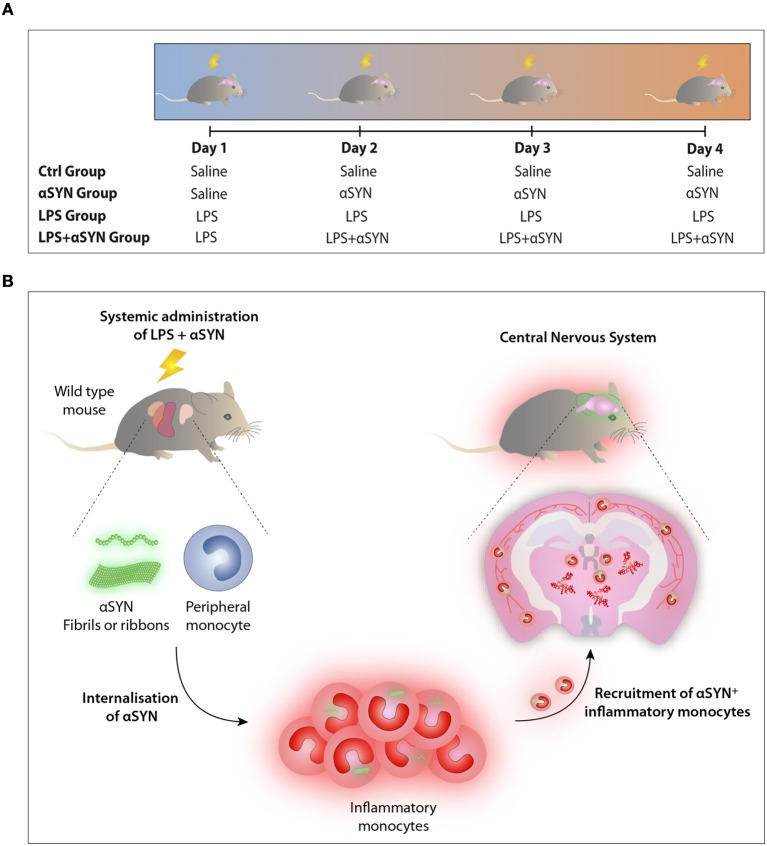
LPS/αSYN administration scheme. **(A)** Mice were treated i.p. with 20 μg of LPS, i.v. with 5 μg of either two atto-488-labeled pathogenic αSYN strains (fibrils or ribbons), with LPS combined with αSYN as aforementioned, or with saline accordingly and following the administration scheme depicted. Twelve hours after the last injection, mice were euthanised and immune cells were isolated from either whole brain or spinal cord and stained for subsequent flow cytometric analysis. **(B)** Proposed model: Inflammatory monocytes, from pivotal sentinels to potential Trojan horses driving the dissemination and propagation of αSYN toward the CNS.

Having identified inflammatory monocytes as potential disease-modifiers, we believe it is necessary to understand the mechanism underlying the internalization and transmission of αSYN as well as its interplay with T cells. Based on our previous report demonstrating that type I interferons are required to induce the selective migration of inflammatory monocytes upon peripheral inflammation (8), targeting these cytokines might hinder the recruitment of these cells and therefore ameliorate the outcome of synucleinopathies like PD. Overall, our findings demonstrate that systemic inflammation induces the recruitment of peripheral leukocytes into the CNS, suggesting that inflammatory monocytes could be turning from pivotal sentinels into potential Trojan horses driving the spreading and propagation of αSYN during disease progression (Figure 2B).

## Materials and Data Availability Statement

The datasets used and/or analyzed during the current study are available from the corresponding author on reasonable request.

## Author Contributions

JPR conceived and designed the research study, performed the experiments, analyzed data, and wrote the manuscript. PI conceived the research study and aided in interpreting the results. LB generated αSYN strains. RM discussed and commented on the manuscript. VB discussed and commented on the manuscript. AVDP designed the research study, performed the experiments, wrote and supervised the manuscript. All authors reviewed the manuscript before submission.

### Conflict of Interest Statement

The authors declare that the research was conducted in the absence of any commercial or financial relationships that could be construed as a potential conflict of interest.
